# Stroke history higher in asymptomatic versus symptomatic atrial fibrillation patients

**Published:** 2018

Newly diagnosed asymptomatic atrial fibrillation patientshave a higher rate of previous stroke than those withsymptoms, according to results from the GLORIA-AFregistry presented recently at EHRA Europace – CardioSTIM2017. The findings highlight the need for screening to identifyatrial fibrillation patients with no symptoms so that strokeprevention treatment can be given.

‘Patients with non-valvular atrial fibrillation have a fivefoldincreased risk of stroke compared to those withoutatrial fibrillation,’ said lead author Dr Steffen Christow,a cardiologist at Hospital Ingolstadt GmbH, Ingolstadt,Germany. ‘Strokes in patients with non-valvular atrialfibrillation tend to be particularly severe and disabling, withabout half of patients dying within one year.’

‘Appropriate anticoagulant therapy substantially reduces the risk of stroke, but in many cases non-valvular atrial fibrillation is only diagnosed after a patient has had a stroke,’ he continued. ‘When patients are unaware of their atrial fibrillation they remain untreated and unprotected from stroke.’

GLORIA-AF (Global Registry on Long-Term Oral Antithrombotic Treatment in Patients with Atrial Fibrillation) is a large, multinational, prospective registry programme involving patients with newly diagnosed non-valvular atrial fibrillation. This sub-analysis compared characteristics between symptomatic and asymptomatic patients in Western Europe.

The study included 6 011 consecutively enrolled patients with non-valvular atrial fibrillation in Western Europe. Symptom status was defined by the European Heart Rhythm Association (EHRA) score: I–II asymptomatic/minimally symptomatic; III–IV symptomatic.

A total of 4 119 patients (two-thirds) were asymptomatic/ minimally symptomatic (hereafter referred to as ‘asymptomatic’) and one-third (1 892) were symptomatic at the time of diagnosis. A number of differences were observed between the two groups.

In terms of medical history, asymptomatic patients were twice as likely to have permanent atrial fibrillation (15.8 vs 8.3%) and more than twice as likely to have had a previous stroke (14.7 vs 6.0%) than patients in the symptomatic group. Asymptomatic and symptomatic patients had a similar number of stroke risk factors, as indicated by a CHA2DS2- VASc score of 3.3 in each group.

Dr Christow said: The finding of a higher rate of previous stroke in the asymptomatic patients despite no differences in the number of stroke risk factors may be explained by a longer but undiagnosed history of atrial fibrillation.’

‘Our study found that in Western Europe, two-thirds of patients newly diagnosed with atrial fibrillation were asymptomatic,’ he continued. ‘Without detection, patients may not receive appropriate preventive therapy and remain at increased risk of stroke.’

Dr Christow concluded: ‘These results underline the urgent need for public programmes to detect atrial fibrillation in the general population.’
